# Asparagus Roots: From an Agricultural By-Product to a Valuable Source of Fructans

**DOI:** 10.3390/foods11050652

**Published:** 2022-02-23

**Authors:** Isabel Viera-Alcaide, Amel Hamdi, Rafael Guillén-Bejarano, Rocío Rodríguez-Arcos, Juan Antonio Espejo-Calvo, Ana Jiménez-Araujo

**Affiliations:** 1 Food Phytochemistry Department, Instituto de la Grasa, Consejo Superior de Investigaciones Científicas (CSIC), Pablo de Olavide University Campus, Building 46, Carretera de Utrera Km 1, 41013 Seville, Spain; iviera@ig.csic.es; 2 Phytochemicals and Food Quality Group, Instituto de la Grasa, Consejo Superior de Investigaciones Científicas (CSIC), Pablo de Olavide University Campus, Building 46, Carretera de Utrera Km 1, 41013 Seville, Spain; amelhamdi1988@yahoo.fr (A.H.); rguillen@ig.csic.es (R.G.-B.); rrodri@ig.csic.es (R.R.-A.); 3 Tecnofood I+D Solucions S.L., 18100 Granada, Spain; jaespejo@hotmail.com

**Keywords:** asparagus by-product, roots and rhizomes, inulin, fructooligosaccharides, asparagus variety, degree of polymerization, structural characteristics, circular economy, sustainability

## Abstract

Asparagus roots are by-products from asparagus cultivation and they could be considered one of the best sources of fructans. These polymers are interesting food ingredients for their prebiotic and immuno-stimulating characteristics. The aim of this work is to characterize the fructan profile from the roots of several asparagus varieties grown at different locations and pickled at three vegetative statuses in order to valorize these by-products as fructan source. Fructans were extracted with hot water and fractionated into three pools according to their molecular weight (MW). Their average MW was studied by HPSEC and their degree of polymerization by HPAEC. The fructan content was up to 12.5% on fresh weight basis, depending on variety and sampling date. The relative abundance of the three pools also depended on the picking moment as after the spear harvest period their total content and MW increased. The average MW of the three fractions was similar among varieties with 4.8, 8.4 and 9 sugar units, although fructans up to 30 units were identified by HPAEC. These characteristics make them similar to the commercialized Orafti^®^-GR inulin, a common additive to food products. Therefore, the concept of asparagus roots as cultivation waste must be changed to a new feedstock for sustainable agriculture and industry.

## 1. Introduction

Asparagus plantations have to be renewed after 8–10 years’ exploitation due to an important reduction of their productivity. Roots and rhizomes are usually dug out, cut mechanically and left on fields as they are considered just cultivation by-products. However, this agricultural practice causes other problems such as the dissemination of allelopathy [1], fungal infections and, finally, asparagus decay [2]. This by-product accounts for up to 30–40 Tm/ha and, taking into account that 1.6 Mha were intended for asparagus cultivation worldwide in 2019 [3], around 6.5 MTm asparagus roots are produced yearly, which becomes a great environmental challenge.

The presence of fructans in the *Asparagus* genus has been investigated from a long time ago [4,5,6], but very little has been done to consider this agricultural product as a fructan source [7,8]. As with other overwintering plants, the fructans in the asparagus plant are accumulated in the roots and rhizomes [9,10,11]. This fact points to these organs as probable fructan sources that have to be characterized for being valorized.

Fructans are basically composed of fructose (F) units linked to glucose (G) in sucrose, but several structures have been defined: inulin-type fructans with β(2→1) linkages [G-β(2→1)-F-β(2→1) -(F)n], levan-type fructans with β(2→6) linkages [G-β(2→1)-F-β(2→6)-(F)n], inulin neo-series where fructose is bonded β(2→6) to glucose in sucrose and β(2→1) on the fructose residue [(F)n-β(2→1)-F-β(2→6)-G-β(2→1)-F-β(2→1)-(F)n], and levan neo-series where fructose is linked β(2→6) to glucose in sucrose and β(2→6) on the fructose residue [(F)n-β(2→6)-F-β(2→6)-G-β(2→1)-F-β(2→6)-(F)n] [12]. Inulin-type fructans are accumulated in Asteraceae plants such as chicory (*Cichorium intybus* L.) and Jerusalem artichoke (*Helianthus tuberosus* L.), while a mixture of inulin and its neo series are found in the lily family, such as onion (*Allium cepa* L.), garlic (*Allium sativum* L.), agave (*Agave* spp.) and asparagus (*Asparagus* spp.). Levan and levan neo-series are more frequent in cereals and grasses [12,13]. The degree of polymerization (DP) varies from 3 to 200. Usually, polymers with DP 2–4 are considered fructooligosaccharides (FOS), those up to DP 7 as oligofructose, and the term “inulin” is applied to higher DP [14].

For commercial purposes, the industrial production of fructans depends on the required DP. FOS are synthetized by biotechnological means, using suspended or immobilized microorganisms or, more frequently, isolated enzymes, whether free or immobilized [14,15]. Oligofructose and inulin are obtained from natural sources, mainly chicory, although other sources are being exploited, such as blue agave and Jerusalem artichoke [15].

In humans, the consumption of inulin-type fructans provides great health benefits, including the regularization of bowel functions, reduction in cholesterolemia and triglyceridemia, resistance to common infections, improved Ca/Mg absorption, balance of colonic microflora and regulation of appetite [16]. Because they are considered as dietary fiber, they can be included in food formulations and labeled as ingredients but not as additives. Consequently, they are widely used as fat and sugar replacers in the formulation of low-calorie food products [17]. Nowadays, bakery or meat products, dairy, breakfast cereals, jams and juices and even confectionery are easily added with fructans, with the double benefit of enhanced organoleptic characteristics and healthy nutritional composition [15,18].

In this context, the aim of this work is to study a broad range of asparagus roots and rhizomes from distinct Spanish locations, at different harvest dates and of several asparagus varieties in order to have an overall picture of the fructan content in asparagus roots and to determine the influence of the different factors. The obtained fructans will be fractionated, and their molecular weight and structural characteristics determined to discuss their possible technological applications. This information will be crucial to value this by-product as a tentative fructan source. A positive assessment would be of great interest for asparagus producers since it would increase their economic returns and would meet an environmental challenge at the same time.

## 2. Materials and Methods

### 2.1. Plant Material

The samples were picked at two different locations, from four asparagus varieties, and over three moments during the asparagus cultivation cycle. A summary of the different factors is presented in Table 1. The underground organs of asparagus plants (roots and rhizomes) were collected at the experimental fields of IFAPA-Rancho de la Merced in Chipiona (Cádiz, Spain) (36.7475612 N–6.4045746 O), and at several asparagus farms in Huétor-Tájar (Granada, Spain) (37.1872700 N–4.0585510 O). Both locations have different geographical and climatic characteristics. Chipiona (CA) is in a seaside area, with mild summers and winters, a temperature range 11.7–26.1 °C, and a daily thermal oscillation of 8.5 °C [19]. However, Huétor-Tájar (GR) has a more continental climate, with dry and hot summers and cold winters, a temperature range of 1.9–32.5 °C, and a thermal oscillation of 17.1 °C [19]. In CA, the roots of two different varieties were collected, Herkolim (HK) and Primems (PM), both from Limgroup (Horst, The Netherlands). In GR, Grande (GD) asparagus hybrid (Walker Brothers Inc., Elmer, NJ, USA) and a local landrace called “Espárrago de Huétor-Tájar” (HT), a protected geographical indication, were chosen. HK, PM and GR are bred hybrids of *Asparagus officinalis* L., but HT is considered a natural hybrid between the cultivated *A. officinalis* and the wild species *Asparagus maritimus* L. Mill [20].

In both locations, CA and GR, samples were taken at three different vegetative statuses of asparagus plants, when the harvest of plant material did not disturb the asparagus agricultural practices: after asparagus shoot season (June, S1), after the summer period (September, S2), and at the end of autumn, after cutting the ferns (December, S3).

Roots and rhizomes were dug up in different areas of the asparagus fields, always from the central zones, avoiding the marginal ones. The distance between plants in a row was 30 cm and between two adjacent rows was approximately 1 m. Samples of around 10 kg each were collected. All samples were sent to our labs, washed very carefully to remove soil and other undesirable material from the roots and rhizomes, and then left to dry at room temperature and frozen at −20 °C until analysis.

### 2.2. Fructan Extraction

50 g of frozen sample in duplicate were homogenized in a Thermomix^®^ model TM31 (Vorwerk, Spain) with 400 mL of hot water (80 °C) to avoid enzymatic activity. After 30 min in a shaking water bath set at 80 °C and 60 rpm, the samples were filtered through paper filter. The slurry was extracted again under the same conditions. Both filtrates were mixed and assayed for their contents in total sugars and fructans. The root extracts were stored at −20 °C and after defrosting prior to analysis, they were heated to 80 °C and let to cool to room temperature.

Total sugars were determined by the anthrone method [21] after preparing the suitable dilution for each sample. The results were expressed as g/100 g fresh sample.

The total content in fructans was analyzed by the K-FRUC assay Kit from Megazyme (Bray, Ireland). The complete method is described in detail on the Megazyme website [22]. The results were expressed as g/100 g fresh sample.

### 2.3. Purification and Fractionation of Asparagus Fructans

**Figure 1:**
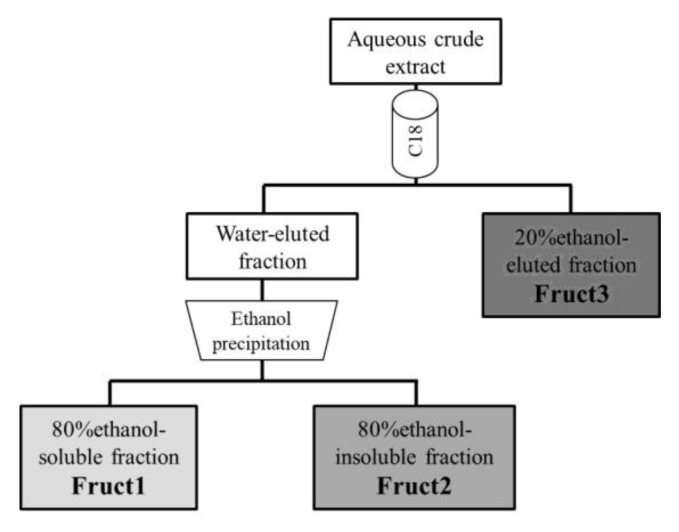
Fractionation of fructans.

Aqueous extracts from the roots were purified using Sep-Pak C18 SPE cartridges (360 mg sorbent per cartridge, 55–105 µm) from Waters (Milford, MA, USA). Prior to use, the cartridges were activated with 96% ethanol and rinsed with water. A diagram of fructan purification and fractionation is presented in Figure 1.

For purification, 5 mL aliquots were slowly loaded into a cartridge and then it was washed with 2 mL distilled water to elute any non-retained material. Afterwards, the cartridge was washed with 5 mL of 20% ethanol. Water and 20%-ethanol fractions were analyzed for fructan content.

For fructan fractionation, the water-eluted fraction was freeze-dried and redissolved in 1 mL water in a screwcap tube. 4 mL 96% ethanol were added and the samples were left overnight at 4 °C. The ethanol-insoluble material was recovered by centrifugation, and the ethanol-soluble fraction was concentrated, lyophilized and assayed for fructan content (Fruct1). The fructan content of the ethanol-insoluble fraction (Fruct2) was calculated by subtracting the fructans present in Fruct1 from the total quantified in the water-eluted fraction. The fructans present in the 20% ethanol-eluted fraction were called Fruct3, as illustrated in Figure 1.

### 2.4. Study of Average Molecular Weight Distribution by Gel Filtration

The three different pools of fructans (Fruct1, Fruct2, and Fruct3) were studied by high performance size-exclusion chromatography (HPSEC) as described in Dos-Santos et al. [23]. The average molecular weight (MW) was measured in a Jasco equipment (LC-Net II ADC, Kyoto, Japan) with a refractive index detector (Jasco RI-1530) and injection valve (Rheodyne, loop 20 μL, Cotati, CA, USA). A TSKgel G3000PWXL column (300 × 7.8 mm i.d., Tosoh Bioscience GmbH, Griesheim, Germany) was used after calibration with 70, 40, 6 kDa, maltotriose, sucrose and glucose (Fluka, Buchs, Switzerland). The elution was performed at a flow rate of 1 mL/min. The regression equation to calculate the average MW of fructan pools was:

(1) $$t_{elut}=-1.614logMW+16.681,$$

with a R^2^ value of 0.9918. The average DP was calculated by dividing the calculated *MW* by 162.

### 2.5. Study of Polymerization Degree of the Different Fructan Fraction by HPAEC

The samples were analyzed in a Dionex (Sunnyvale, CA, USA) high-performance anion-exchange chromatograph (HPAEC) using a Carbopac PA-10 column (4 × 250 mm, 10 μm) in combination with a Carbopac PA guard column (4 × 50 mm, 10 μm) as described by Jaramillo-Carmona et al. [24]. The mobile phase consisted of 100 mM NaOH (eluent A) and 100 mM NaOH and 700 mM sodium acetate (eluent B). The elution conditions were as follows: 0–15 min, 100% A (re-equilibration); injection at 15 min and start acquisition at 16 min; linear gradient over 55 min to 20% A, 80% B. The flow rate was maintained at 0.9 mL/min. A Dionex pulsed electrochemical detector in the pulsed amperometric detection (PAD) mode was used. The calibration for the different DP was done using a commercial fructan from chicory, Orafti^®^GR (Beneo GmbH, Germany), with an average DP ≥10 [25].

### 2.6. Study of Fructan Structure by Methylation (GC-MS)

The water- and 20% ethanol-eluted fractions were methylated according to a modification of the Hakomori method [26]: 0.1 mL of butyl-lithium, 15% in hexane was added to samples dissolved in 0.4 mL of dimethyl sulfoxide. The mixture was then shaken for 1 h at 40 °C [27]. After the samples had been frozen, 0.2 mL of ethyl iodide was added, and the samples were allowed to thaw at room temperature and later shaken for 1 h. The permethylated polysaccharides were separated and purified by reverse phase chromatography using a Sep-Pak. The polysaccharides were hydrolyzed, reduced, and acetylated [28]. The partially methylated alditol acetates were separated by GC/mass spectrometry (GC/MS) in an Agilent technology 7820A instrument, which was coupled to an Agilent 5977E selective mass detector with a fused silica capillary column (30 × 0.25 m, SPTM 2330, Supelco^®^, Sigma-Aldrich, St. Louis, MO, USA) in splitless mode. The oven temperature program was the same as the one used by York et al. [28]. For quantification, molar effective response factors were used [29].

### 2.7. Statistical Analysis

All samples were analyzed at least in triplicate. To assess the differences among samples, a multiple sample comparison was performed using the Statgraphics^®^ Plus program Version 2.1. The level of significance was *p* < 0.05.

## 3. Results and Discussion

### 3.1. Contents in Neutral Sugars and Fructans

The neutral sugar and fructan contents were analyzed in the initial aqueous extract from the twelve samples. The results for neutral sugars are given in Table 2. The content varied from nearly 8% (HK-S3 and GD-S2) to 26% (HK-S2), but no common behavior could be found among samples: PM and GD had the lowest contents in S2, S1 and S3 without showing significant differences; however, the HK content increased in S2 and decreased in S3, and HT only increased in S3.

In relation to fructan contents (Table 3), the lowest level was found in HT-S1, at nearly 1%, and the highest in HK-S2 (the same sample with the highest sugar level), which was higher than 12%. The fructan range was much broader than that of sugar, showing a great variability among samples. The average value was 5.33% f.w. This result was lower than those found in the crops specifically devoted to fructan production: 11–14% for Jerusalem artichoke [30], 15–20% for chicory [31], and higher than 37% for agave (*Agave tequilana* Weber var. Azul) [32]. However, it is important to note that asparagus roots are by-products that suppose agricultural and environmental challenges and to find an alternative use could be of great interest. By choosing the optimal harvest moment, this percent could increase and the fructan extraction would suppose an added value for asparagus spear producers. Another factor to be studied was the ratio between total neutral sugars and fructans. The percent of fructans in the total sugars for the different varieties and samplings is presented in Table 3 (bold numbers). In all varieties, S2 and/or S3 showed better results than S1. From this point of view, both picking moments could be the most appropriate for fructan extraction due to their lower contents in free sugars. Therefore, roots harvested during autumn or winter could bring asparagus extracts which are richer in fructans than those from the summer harvest. In addition, neither moment would interfere in the normal asparagus cultivation practices.

Looking at the different varieties, HK was the richest one in fructans and PM the poorest. It is interesting to note that both varieties were collected in CA and at the same experimental fields, so variety genetics play an important role in fructan synthesis, accumulation, mobilization, and degradation, which is also true for other fructan-synthetizing crops e.g., agave [33], onion [34] and chicory [35]. In asparagus spears, the influence of variety was previously observed in the accumulation of secondary metabolites such as flavonoids [36,37] and saponins [38,39].

The differences between the harvest moments were significant in most cases, and only PM variety did not show differences between S1 and S2 or S3. Only HT showed a clear increasing tendency in fructan content (Table 3). This differential behavior remarked the decisive influence of variety on fructan metabolism.

Another fact to point out is the difference between S2 and S3. In samples from CA (HK and PM), significant decreases were quantified in fructan contents but in contrast, the opposite behavior was found in samples from GR (GD and HT). This could be due to different cultivation practices and/or climatic conditions. 

### 3.2. Fructans Fractionation

**Figure 2:**
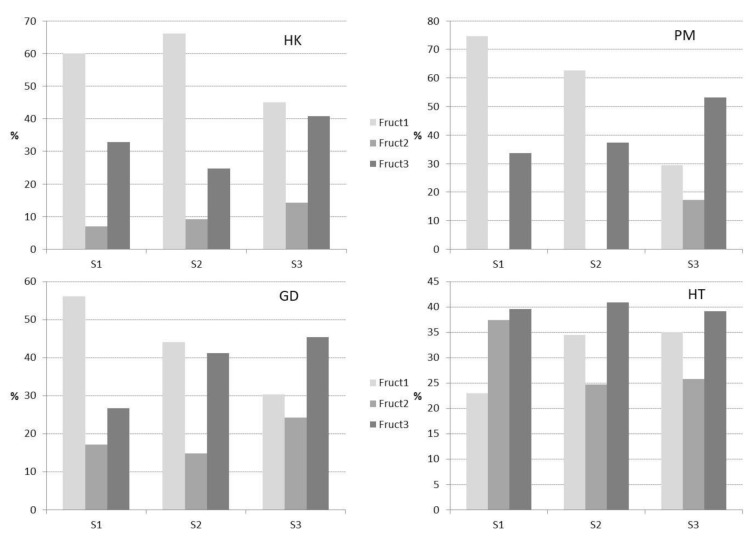
Relative percentages of the different fructan fractions of the different studied fractions. S1: samples picked in June; S2: samples picked in September; S3: samples picked in December; GD: Grande variety; HK: Herkolim variety; HT: Huétor-Tájar landrace; PM: Primems variety; Fruct1: fructan fraction soluble in 80% ethanol; Fruct2: fructan fraction insoluble in 80% ethanol; Fruct3: fructan fraction eluted from C18 cartridge with 20% ethanol.

Each extract was fractionated as presented in Figure 1 and the three obtained fractions were analyzed for their contents in fructans (Figure 2). HK, PM and GD showed similar results: Fruct1 was the major fraction in S1 and S2; in HK-S3, Fruct1 and Fruct3 were in similar proportions but in PM-S3 and GD-S3, Fruct3 was clearly the major one. It seems that the plant has metabolized most of its fructan at S1, with those of the lowest MW being the most abundant. As the season progressed (S2 and S3), the fructan stock shifts to higher MW, increasing the percentages of Fruct2 and specially those of Fruct3.

However, the behavior of the HT variety was again different. At S1, Fruct1 was the minority pool, with Fruct2 and Fruct3 in similar proportions. S2 and S3 had the same profile, although the total amount increased from S2 to S3 (Table 3). It seems that the partly-wild character of this variety also marked differences in the pattern of synthesis and accumulation of fructans. This variety was also clearly different when studying some secondary metabolites (flavonoids and saponins). HT spears are richer in both groups of phytochemicals than those of other *A. officinalis* varieties, and also present a higher variability in their chemical structures [36,37,38].

In relation to harvest time, similar results were found by Suzuki et al. [10] while working with different types of asparagus storage roots. They concluded that the fructan MW from winter-collected roots was higher than that from summer-collected ones. They also observed that new roots accumulated fructans with higher MW than the oldest ones, pointing to a lower activity of elongating enzymes (fructan:fructan 1-fructosyltransferase, 1-FFT) in mature roots. 

But harvest time is not the only factor that could affect fructan chain length, apart from the genetic ones. In Jerusalem artichoke tubers, prolonged storage periods at 5 °C led to shorter polymers than those extracted from roots stored at 2 °C or frozen tubers [40]. Working with wild agave varieties with different ages (2–12 years-old), Aldrete-Herrera et al. [33] observed that the oldest plants (10–12 years-old) had the lowest concentrations of free sugars and the highest of high MW fructans. These results are in contrast to those presented above for different asparagus roots, again highlighting the significance of plant genetics. Growth climatic conditions also influence the total content and length of fructans. In fact, drought increases both parameters in chicory [41]. The paramount importance of fructans in lipid bilayer stabilization against abiotic stresses is well known, providing the plants with drought, freezing and/or chilling tolerance [42]. From all these works, the great complexity of fructan biosynthesis, elongation and degradation pathways is clear along with the variety of factors that could influence all these processes. The characterization of the raw material (asparagus root by-product in the present work) is a crucial step for the knowledge of the factors that influence the final contents and characteristics of fructans, key parameters for the valorization of this by-product.

### 3.3. Molecular Weight Distribution and Structural Features

**Figure 3:**
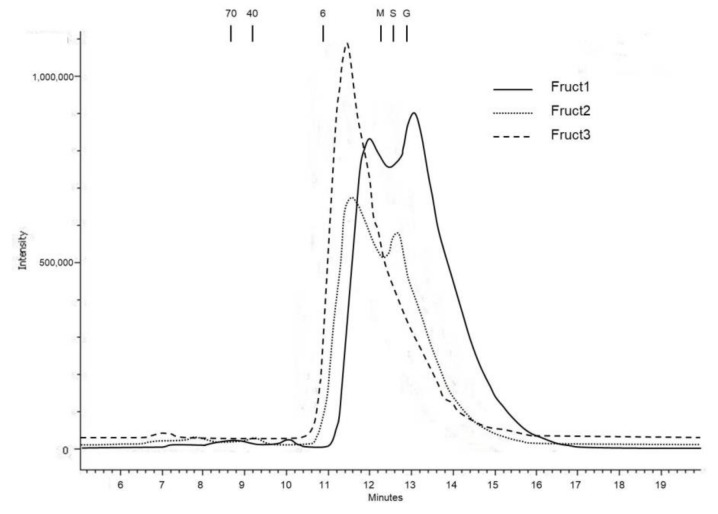
HPSEC-RI profiles of the three fructan fractions from HK-S2 sample. HK-S2: sample of Herkolim variety picked in June; Fruct1: fructan fraction soluble in 80% ethanol; Fruct2: fructan fraction insoluble in 80% ethanol; Fruct3: fructan fraction eluted from C18 cartridge with 20% ethanol; 70: dextran standard 70 kDa MW; 40: dextran standard 40 kDa MW; 6: dextran standard 6 kDa MW; M: maltotriose; S: sucrose; G: glucose.

The first approach to the MW study was done by HPSEC. In Figure 3, the three fractions corresponding to HK-S2 are presented. Fruct1 and Fruct2 eluted in two differentiated pools. In order to identify the presence of fructans, fractions were collected and assayed for fructan existence. They were only detected at the first-eluted peak, with the second one being composed probably of sucrose, fructose and glucose, as observed in the elution time of standards. In the case of Fruct1, the first peak eluted with a maximum at 12 min and 11.6 min for Fruct2. Fruct3 showed a single peak with its maximum at 11.55 min. The average DP was calculated by equation (1) as 4.8, 8.4 and 9 for Fruct1, Fruct2 and Fruct3, respectively. HK-S1 and HK-S3 showed the same elution maxima, but the relative intensity of the three fractions changed according to the results presented in Figure 2. The rest of the varieties showed very similar profiles. These MWs were much lower than those published for other vegetable products, especially those considered as fructan sources: from agave, fructans with an average DP of 29 have been described [32], and even higher from artichoke by-products, average DP 42–46 [43,44]. Similar results have been found for chicory roots with an average DP of 14 [45].

**Figure 4:**
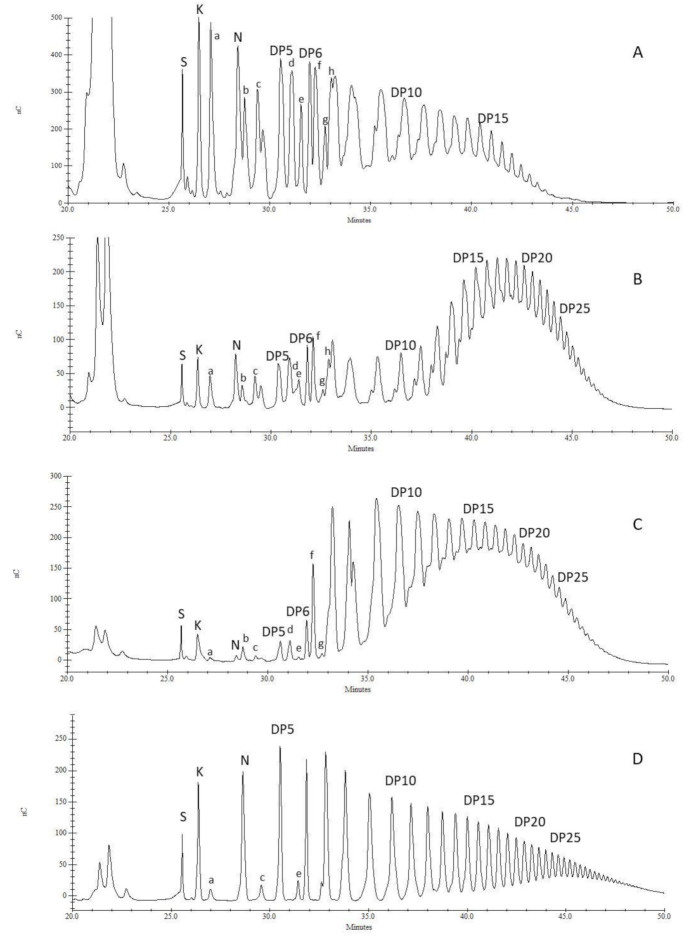
HPAEC profiles of the three fructan fractions from the HK-S2 sample and Orafti^®^-GR as DP standard. (**A**): Fruct1 (fructan fraction soluble in 80% ethanol); (**B**): Fruct2 (fructan fraction insoluble in 80% ethanol); (**C**): Fruct3 (fructan fraction eluted from C18 cartridge with 20% ethanol); (**D**): Orafti^®^-GR; HK-S2: sample of Herkolim variety picked in June; S: sucrose; K: kestose; N: nystose; DP: degree of polymerization of inulin-series compounds; a-h peaks: inulin neo-series compounds as tentatively identified according to Witzel and Matros [11].

To go deeper into DP determination, we studied all samples by HPAEC. The obtained profiles for Fruct1, Fruct2 and Fruct3 from HK-S2 are presented in Figure 4, and also the profile of a commercial chicory inulin approved as dietary fiber for human nutrition (Orafti^®^-GR from Beneo). Orafti^®^-GR was included in the study to obtain standards for the different DPs. There are clear differences among fractions. In Fruct1, the highest peak population was around DP5. As it was previously described by other authors, asparagus fructans are from both inulin-type and inulin neo-series [9,10,46]. Suzuki et al. [10] determined that the total number of isomers for a DP value of n is n−1. These facts could explain the great number of compounds isolated between sucrose and DP10 from asparagus roots (Figure 4), especially in Fruct1 fraction, in comparison with the chicory fructan Orafti^®^-GR, presented as standard. Shiomi et al. [5,6] and Shiomi [4,9] have described up to 14 different isomers of DP 4-8 from the roots of *A. officinalis*, belonging to both inulin and neo series. Witzel and Matros [11] and Matros et al. [47] analyzed asparagus root and barley fructans by mass spectrometry, identifying each compound based on fructanase treatment and mild acid hydrolysis. According to these works, the compounds named a-h in Figure 4 were tentatively identified. Sucrose (S), kestose (K, DP3), nystose (N, DP4) and peaks called DP5-25 were identified by their retention times compared to standards (S, K, N as standard compounds and the rest with Orafti^®^-GR). Therefore, the peaks corresponding to the inulin series [G-β(2→1)-F-β(2→1)-(F)n] were named DP5-25, and those of the inulin neo series [(F)n-β(2→1)-F-β(2→6)-G-β(2→1)-F-β(2→1)-(F)n] were named a-h (Figure 4). This last group was identified according to Witzel and Matros [11].

Compound “a” was neo-kestose [F-β(2→6)-G-β(2→1)-F] and “b” and “c” two neo-nystoses [F-β(2→6)-G-β(2→1)-F2 and F2-β(2→6)-G-β(2→1)-F, respectively]. In peak “d”, two different compounds coeluted both tentatively corresponding to neo-DP5, F-β(2→6)-G-β(2→1)-F3 and F2-β(2→6)-G-β(2→1)-F2. Another neo-DP5 was identified in peak “e”, F3-β(2→6)-G-β(2→1)-F. Peak “f” corresponds to a mixture of three neo-DP6, F-β(2→6)-G-β(2→1)-F4, F2-β(2→6)-G-β(2→1)-F3 and F3-β(2→6)-G-β(2→1)-F2. Compound “g” could be tentatively assigned to the last neo-DP6 isomer F4-β(2→6)-G-β(2→1)-F. From this MW and higher, the identification of the different polymers is more difficult mainly due to coelution and to a decrease in the detection limit [11]. Therefore, peak “h” could tentatively correspond to the coelution of four different isomers with DP7, inulin series and three neo series (F-β(2→6)-G-β(2→1)-F5, F2-β(2→6)-G-β(2→1)-F4 and F4-β(2→6)-G-β(2→1)-F2). Some of these oligosaccharides were previously reported in the roots of *A. officinalis* and *A. racemosus* [4,5,6,9,48]. The rest of the samples had similar distributions, only showing slight differences in the relative abundance of the different peaks. The samples corresponding to HT showed a different behavior in fructan accumulation throughout the season (Table 3) and during Sep-Pak fractionation (Figure 2). However, their HPAEC profiles were similar to those presented for HK-S2 (Figure 4).

In order to discriminate the proportion of inulin and inulin neo-series in asparagus roots, a methylation analysis was performed and the sugar derivatives were identified by GC-MS (data not shown). In all analyzed samples, the ratio between 6-glucose (inulin series) and 1,6-glucose (inulin neo-series) was 1:2, suggesting that the fructans from the neo-series were twice the inulin one.

Peaks from “a” to “h” were more abundant in the Fruct1 fraction (Figure 4A), decreasing their proportion in Fruct2 and Fruct3 (Figure 4B,C). In the last one, most polymers isolated had DP ≥ 7. Some of these peaks (a–h) are also present in Orafti^®^-GR in trace amounts (Figure 4D). In Fruct1, fructans with DP up to 15–20 were identified in smaller amounts. However, in Fruct2 and Fruct3, higher DP oligomers were found (DP 25–30). This fructan size is slightly higher than described by other authors [10,11,46] where polymers up to 21 units were reported in asparagus roots.

In the literature, other fructan sources or agricultural byproducts have been characterized for their fructan DP. Similar results to asparagus roots were found in Jerusalem artichoke tubers [40]; however, those from other Asteraceae species (*Cynara cardunculus* L. and chicory) were described up to 200 and 123, respectively [45,49]. In *Agave* spp. a wide range of DP was found among different species and varieties, with the maximum DP up to 70 [33]. It is important to keep in mind that all these results depend greatly on a series of genetic, environmental and physiological factors which could modify the average and the highest fructan DP to a great extent [42,49]. It is interesting to note that fructans characterized in this work from asparagus roots are very similar to Orafti^®^-GR from Beneo, which is widely applied in most food and drink formulations ((baked and baby foods; breakfast cereals and bars; candy and chocolates; dairy and meat products; soups, sauces and fillings; etc.) [25]. This fact could point to possible applications for asparagus fructans in human nutrition as fat replacer as they could have a creamy taste and similar mouthfeel to fat, but with reduced caloric value and improved stability. They are also highly soluble in water, so they could be added in high dosages without adapting or changing the different production processes [25].

## 4. Conclusions

The underground organs of asparagus plants from two locations and several varieties and sampling dates have been investigated for their content in fructans. Asparagus variety and sampling period are the most influential factors. The last one must be optimized in order to reach the highest fructan yield, but it is clear that the best picking period could be from the end of summer to winter. In this time period, the roots have recovered their fructan stock which was depleted during spring sprouting. The range of fructan content was 3–12% in fresh weight basis (8–33% in dry weight basis). Our highest level is lower than those published for other fructan sources (chicory, Jerusalem artichoke and agave) but very close to them. Therefore, if harvested at their optimal moment, asparagus roots and rhizomes could be considered a promising starting point for fructan isolation. In most studied varieties, both the total amount of fructans and the content of those with the highest MW increase during summer and autumn. In our investigations, the DP of asparagus root fructans was up to 25–30 sugar units. Therefore, they can be considered as inulin. In addition, a great variety of oligomers of DP 3–10 were also described. Therefore, FOS (DP 2–5) and oligofructose (up to DP 7) were also present in asparagus root fructans. The relative percent of each fraction varied during the rest period of asparagus plants.

With this work, the interest in the underground organs of asparagus as a source of fructans has been established. Further studies about extraction optimization, fructan purification and potential applications will be required in order to give a practical approach to this initial research. These achievements will provide compelling reasons to change the farming practices of asparagus growers, which would alleviate the environmental challenge of asparagus cultivation. 

## Figures and Tables

**Table 1:** Sampling of underground organs of asparagus.

Location	Variety	Species	Sampling Date
Chipiona (Cádiz) CA	Herkolim HK	*A. officinalis*	June S1 September S2 December S3
	Primems PM	*A. officinalis*	
Huétor-Tájar (Granada) GR	Grande GD	*A. officinalis*	
	Huétor-Tájar landrace HT	*A. officinalis* × *A. maritimus*	

**Table 2:** Sugar content (g/100 g fresh weight) of the different asparagus root samples.

	S1	S2	S3
Herkolim	13.41 ± 0.99 c	26.58 ± 1.75 g	8.34 ± 0.76 a
Primens	14.84 ± 0.73 d	11.23 ± 0.81 b	15.60 ± 1.11 de
Grande	16.44 ± 1.30 e	8.27 ± 0.69 a	15.95 ± 1.39 de
Huétor-Tájar landrace	11.69 ± 0.50 b	10.49 ± 0.85 b	18.89 ± 1.80 f

All analyses were done at least in quadruplicate. The results are presented as mean ± standard deviation. Means bearing the same letter are not significantly different at the 5% level, as determined by the Duncan multiple range test. S1: samples picked in June; S2: samples picked in September; S3: samples picked in December.

**Table 3:** Fructan content (g/100 g fresh weight) and percent of fructan on total sugars (bold numbers) of the different asparagus root samples.

	S1	S2	S3
Herkolim	6.36 ± 0.20 f **47.47**	12.53 ± 0.44 h **47.13**	5.73 ± 0.21 e **68.70**
Primens	3.08 ± 0.30 bc **20.76**	3.41 ± 0.23 c **30.37**	2.87 ± 0.21 b **18.41**
Grande	5.33 ± 0.49 e **32.43**	4.80 ± 0.39 d **58.07**	6.27 ± 0.56 f **39.33**
Huétor-Tájar landrace	0.92 ± 0.08 a **7.90**	4.70 ± 0.22 d **44.81**	8.02 ± 0.30 g **42.45**

All analyses were done at least in quadruplicate. The results are presented as mean ± standard deviation. Means bearing the same letter are not significantly different at the 5% level, as determined by the Duncan multiple range test. S1: samples picked in June; S2: samples picked in September; S3: samples picked in December.

## Data Availability

Not applicable.
